# Strong and Ethical Scholarly Writing for Multidisciplinary Audiences

**DOI:** 10.1093/geroni/igaa057.3161

**Published:** 2020-12-16

**Authors:** Suzanne Meeks

**Affiliations:** University of Louisville, Louisville, Kentucky, United States

## Abstract

This presentation will emphasize the importance of plain, good writing. Editors of high impact journals read 10 or more manuscripts per week, and are under pressure to reject 80-90% of them. Regardless of scholarly quality, if the point and contribution are not clear in a quick scan of the paper, it likely will not be reviewed favorably. I will provide tips for strong scientific writing that are commonly violated in manuscript submissions, and provide references for additional writing support. I will also discuss some common publication ethics issues that arise during the review process, including author contributions and embedding your scholarship in the context of prior work.

